# Fundamental limits of ultrathin metasurfaces

**DOI:** 10.1038/srep43722

**Published:** 2017-03-06

**Authors:** Amir Arbabi, Andrei Faraon

**Affiliations:** 1 T. J. Watson Laboratory of Applied Physics, California Institute of Technology, 1200 E California Blvd., Pasadena, CA 91125, USA

## Abstract

We present a set of universal relations which relate the local transmission, reflection, and polarization conversion coefficients of a general class of non-magnetic passive ultrathin metasurfaces. We show that these relations are a result of equal forward and backward scattering by single layer ultrathin metasurfaces, and they lead to confinement of the transmission, reflection, and polarization conversion coefficients to limited regions of the complex plane. Using these relations, we investigate the effect of the presence of a substrate, and show that the maximum polarization conversion efficiency for a transmissive metasurface decreases as the refractive index contrast between the substrate and cladding layer increases. Furthermore, we demonstrate that a single layer reflective metasurface can achieve full 2*π* phase shift coverage without altering the polarization if it is illuminated from the higher refractive index material. We also discuss two approaches for achieving asymmetric scattering from metasurfaces, and realizing metasurfaces which overcome the performance limitations of single layer ultrathin metasurfaces.

Metamaterials are artificial materials with electromagnetic properties which are controllable by design. They are generally three dimensional periodic arrays of scatterers with deep subwavelength periods, and their electromagnetic properties can be fully represented by their permittivity and permeability tensors. Metasurfaces are two dimensional counterparts of the metamaterials. They are composed of an array of scatterers with sub-wavelength period which are located on a planar surface. Ultrathin metasurfaces are a class of metasurfaces composed of scatterers which are significantly thinner than the wavelength of the light^1^,^2^. An ultrathin metasurface is generally created by subwavelength patterning of an ultrathin film. The film is usually deposited on a flat substrate, and is rationally patterned for modification of the phase^3^,^4^,^5^,^6^, amplitude^7^, or polarization^8^,^9^,^10^,^11^,^12^ of the transmitted or the reflected light. The main feature that distinguishes the ultrathin metasurfaces from conventional diffractive elements and other types of metastructures is their distinctive principle of operation. Ultrathin metasurfaces cause a discontinuity in the phase of the light that is transmitted through or reflected from them. This phase discontinuity is the result of the interference between the incident wave and the scattered light by the ultrathin scatterers^4^,^5^. This is in contrast to the conventional diffractive elements and other types of metastructures which rely on gradual phase shifts accumulation during light propagation across them. Such metasurfaces have attracted a lot of attention recently and several types of flat diffractive elements such as lenses, axicons, and complex beam shapers^5^,^13^,^14^ have been realized using them.

Materials with plasmonic resonances such as gold and silver are popular choices for the metasurface layer. This is because these materials have large negative permittivity; therefore, even a thin layer of them can scatter the light significantly. One of the well-known drawbacks of using these materials is their substantial absorption loss which limits the efficiency of the diffractive elements. As a result, most of the work in this area has been limited to the near and mid-infrared, terahertz, and microwave wavelengths range where the absorption losses are smaller^2^,^7^,^15^,^16^. Recently, by using the network scattering matrix theory, it has been shown that a special class of ultrathin transmissive metasurfaces which are lossless, symmetrical, and reciprocal cannot provide complete phase control unless they alter the polarization, and their polarization conversion efficiency is limited to 25%^17^,^18^. Here, we show that the transmission, reflection, and polarization conversion coefficients of passive non-magnetic ultrathin metasurfaces satisfy a set of fundamental relations. We demonstrate that these fundamental relations extends the limits previously derived for the special class of metasurfaces to encompass reflective and transmissive metasurfaces, metasurfaces with loss, nonreciprocal metasurfaces, metasurfaces with substrate and cladding, and metasurfaces operating at non-normal incident angles. In particular, we show that full 2*π* phase coverage cannot be achieved using reflective ultrathin metasurfaces unless the metasurface is illuminated from the material with higher refractive index. We also show that the inability of the transmissive metasurfaces for achieving 2*π* phase coverage is conserved for lossy metasurfaces and asymmetric metasurfaces (i.e. when the substrate and cladding have different refractive indices). We also demonstrate that the maximum achievable polarization conversion efficiency depends on the refractive index contrast between the substrate and the cladding layer, and it decreases for transmissive metasurfaces as this refractive index contrast increases, while it increases beyond 25% for reflective metasurfaces illuminated from the material with higher refractive index. We show that the main source of these performance limitations is the lack of phase retardation as the light propagates through the metasurface layer, and we suggest two methods for realization of metasurfaces with higher efficiencies.

## Results

**Figure 1:**
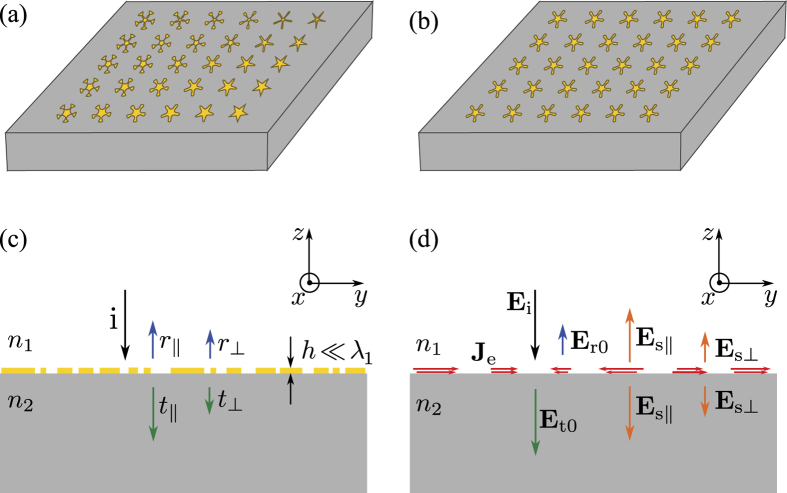
Schematic illustrations of (**a**) a metasurface with gradually varying scatterers, and (**b**) a periodic metasurface. (**c**) Cross section view of the periodic metasurface shown in (**b**). A normally incident plane wave is impinging on the metasurface. The transmitted and reflected plane waves, and the polarization converted plane waves are also shown. (**d**) Illustration of the equivalent volume current density that has replaced the periodic metasurface. **E**_i_, **E**_r0_, and **E**_t0_ are the electric fields of the incident, reflected and transmitted plane waves for a bare interface, respectively. The electric field of the light scattered by the metasurface is labeled by **E**_s||_ for the part with the same polarization as **E**_r0_ and **E**_t0_, and by **E**_s__⊥_ for the part with polarization orthogonal to the polarizations of **E**_r0_ and **E**_t0_.

We consider an ultrathin metasurface which is composed of an array of potentially dissimilar subwavelength passive and non-magnetic scatterers. A schematic illustration of such a metasurface is depicted in Fig. 1(a). The scatterers are resting on a substrate with refractive index of *n*_2_ and are surrounded by a cladding material with the refractive index of *n*_1_. We assume that the scatterers have a thickness of *h* which is much smaller than the wavelength of light in the cladding material (i.e. *h* ≪ *λ*_1_ = *λ*_0_/*n*_1_). The metasurface locally modifies the amplitude, phase, or polarization of an incident light either in transmission or reflection. For weakly coupled and gradually varying scatterers, the metasurface can be modeled as a surface with spatially dependent local reflection and transmission coefficients.

For each of the scatterers comprising the metasurface, we can form a periodic metasurface by arranging that scatterer on a periodic lattice similar to the lattice of the original metasurface in the vicinity of that scatterer. An example of such a periodic metasurface is shown in Fig. 1(b). The reflection and transmission of such a periodic metasurface approximates the local reflection and transmission of the original metasurface at the location of that scatterer. Thus, the properties that we establish for the reflection and transmission coefficients of the periodic metasurface are applicable to the local coefficients of the original metasurface.

We assume that a plane wave is normally incident on the periodic metasurface as shown in Fig. 1(c). Since the period of the metasurface is smaller than the wavelength in both the surrounding materials, only the zeroth order transmission and reflection are propagating waves. In general, the transmitted and reflected light waves do not have the same polarization as the incident light. We decompose each of the transmitted and reflected plane waves into two plane waves with orthogonal polarizations. We represent the transmission and reflection coefficients for the parts of the light with the same polarizations as the plane waves transmitted through and reflected from a bare substrate-cladding interface (e.g. the interface without the metasurface) by *t*_||_ and *r*_||_, respectively. For a linearly polarized light, *t*_||_ and *r*_||_ represent the transmission and reflection coefficients of the parts of the light with the same polarization as the incident light. These coefficients are the total transmission and reflection coefficients for a metasurface that does not modify the polarization. Polarization modification happens if the light reflected from or transmitted through the metasurface acquire some polarization component orthogonal to the polarization of a plane wave reflected from or transmitted through the bare interface. We represent the transmission and reflection coefficients for the parts of the light whose polarization has been converted to the orthogonal polarization by *t*_⊥_ and *r*_⊥_, respectively. We refer to *t*_⊥_ and *r*_⊥_ as polarization conversion coefficients. For example, if the incident plane wave is right handed circularly polarized then *t*_||_ and *t*_⊥_ are the transmission coefficients for the parts of the transmitted light which are right and left handed circularly polarized, respectively, while *r*_||_ and *r*_⊥_ are the reflection coefficients for the parts which are left and right handed circularly polarized, respectively.

Since the materials composing the periodic metasurface are non-magnetic, we can replace the entire metasurface by an equivalent volume electric current density


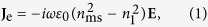


where *ω* is the angular frequency of the incident light, *ε*_0_ is the vacuum permittivity, *n*_ms_ is the complex refractive index of the metasurface materials, and **E** is the total electric field^19^. Note that **J**_e_ is nonzero only at the location of the metasurface, and is confined to the thickness *h* above the substrate as illustrated in Fig. 1(d). According to the volume equivalence theorem, the light emitted by **J**_e_ in the presence of the substrate-cladding interface is equal to the light scattered by the metasurface. The volume current density **J**_e_ is also periodic with the same period as the periodic metasurface it has replaced; therefore, it emits into the substrate and top cladding materials only along the interface normal directions (±z directions in Fig. 1(c and d)).

To find the amplitudes of the plane waves emitted by **J**_e_ into the cladding and substrate, we first find the amplitudes of the plane waves it emits when it is located in the material with the refractive index of *n*_1_. For simplicity, we choose the coordinate system with *z* = 0 plane located at the middle of the metasurface layer; therefore, **J**_e_ is confined to −*h*/2 < *z* < *h*/2 slab region. The electric fields of the plane waves emitted by **J**_e_ along the +*z* and −*z* directions right above (**E**_+*z*_) and below (**E**_−*z*_) the slab region are given by ref. 20


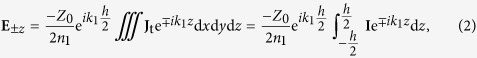


where *k*_1_ = *n*_1_2*π*/*λ*_0_, *Z*_0_ is the impedance of free space, **J**_t_ is the components of **J**_e_ parallel to the *xy* plane, and 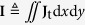. Since |*k*_1_*z*| < *πh*/*λ*_1_ ≪ 1, the 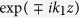 term in (2) can be approximated by the first two terms of its Taylor series expansion


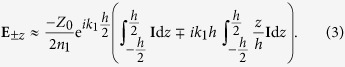


Considering the polarity of the electric fields of the light emitted along ±*z*, we can interpret the first term inside the parentheses in (3) as electric field emitted from an effective surface electric current and the second term as electric field emitted by an effective surface magnetic current. If 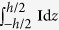 is nonzero then the term corresponding to the surface electric current is the dominant term, and the electric fields of the optical waves emitted by **J**_e_ along the ±*z* directions are equal to each other. As we mentioned earlier, the light does not accumulate significant phase as it propagates through an ultrathin metasurface which operates based on creating a phase discontinuity. Therefore, the phase of the electric field **E** inside the metasurface layer and, according to (1), the phase of **J**_e_ does not vary significantly along the propagation direction inside ultrathin metasurfaces. As a result, 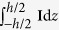 would be nonzero and the amplitudes of the plane waves emitted by **J**_e_ toward the ±*z* directions would be equal to each other.

Some of the light emitted by **J**_e_ toward the −*z* direction is reflected back at the substrate-cladding interface. We obtain the electric field amplitudes of the plane waves emitted by **J**_e_ inside the substrate (**E**_s−_) and cladding (**E**_s+_) as


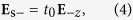



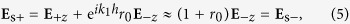


where *r*_0_ = (*n*_1 _−_ _*n*_2_)/(*n*_1_ + *n*_2_) and *t*_0_ = 2*n*_1_/(*n*_1_ + *n*_2_) are respectively the reflection and transmission coefficients of the bare substrate-cladding interface for normally incident plane waves. To obtain (5), we have used exp(*ik*_1_*h*) ≈ 1 and the relation *t*_0_ = 1 + *r*_0_. Since the electric fields of the optical waves scattered by the metasurface into the substrate and the cladding are equal to each other, we omit the + and − subscripts and represent both by **E**_s_. According to the superposition principle^19^, the total transmitted and reflected optical waves are the summation of the waves transmitted through and reflected from the bare substrate-cladding interface, plus the light emitted by **J**_e_. As shown in Fig. 1(d), **E**_s_ is decomposed into two plane waves with orthogonal polarizations. The parts of **E**_s_ which have the same polarizations as 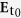 and 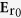 are represented by **E**_s||_, and the parts which have polarizations orthogonal to them are shown by **E**_s⊥_.

Since the periodic metasurface is passive, the sum of the transmitted and reflected powers is equal to, or smaller than (for lossy metasurfaces) the incident power, that is


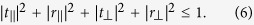


For the bare interface we have


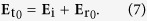


We can express the transmission and reflection coefficients of the periodic metasurface in terms of the electric field amplitudes as


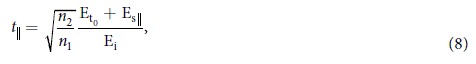



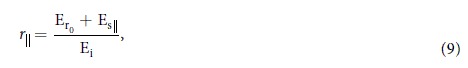



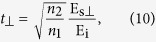



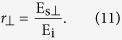


Using (6) to (11), we find the fundamental relations of ultrathin metasurfaces as


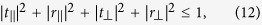



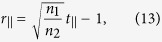



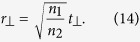


By using a similar procedure, we can show (see Supplementary Information) that when the incident light is incident at an angle *θ*_*i*_ with respect to the interface normal direction, relation (12) is still valid and metasurface relations (13) and (14) are modified as


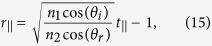



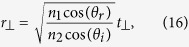


for a transverse electric (TE) polarized incident plane wave, and as


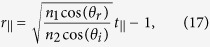



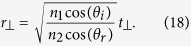


for a transverse electric (TM) polarized incident light. Here, *θ*_*r*_ is the angle of refraction and 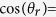 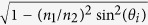.

The transmission and reflection coefficients of any periodic ultrathin metasurface satisfy (12)–(14). As we mentioned earlier, the local transmission and reflection coefficients of a metasurface with gradually enough varying parameters is similar to those of a periodic metasurface without the gradual variations of the metasurface. Therefore, the relations (12)–(14) are valid for the local transmission, reflection and polarization conversion coefficients of an ultrathin metasurface. It should be noted that the limitations expressed by (12)–(14) are merely the results of deep subwavelength thickness of the metasurface, and are valid regardless of the potential absorption loss caused by metasurfaces. Material absorption loss will tighten the limit in (12) even further. More specifically, for a lossy metasurface the left hand side of (12) is equal to 1 − *L*, where *L* is the fraction of the light absorbed by the metasurface.

In the following, as two example cases, we discuss the implications that the relations (12)–(14) have on the performance of reflective and transmissive metasurfaces designed for shaping the phase front, and modifying the polarization of a normally incident light.

### Phase Front Modification with Ultrathin Metasurfaces

Consider an ultrathin metasurface which is designed to shape the phase front of an incident beam to a desired form without disturbing its polarization. For such a metasurface, by using (12) and (13), we find


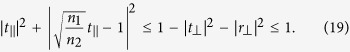


We can also express (19) in terms of reflection coefficient as


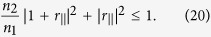


**Figure 2:**
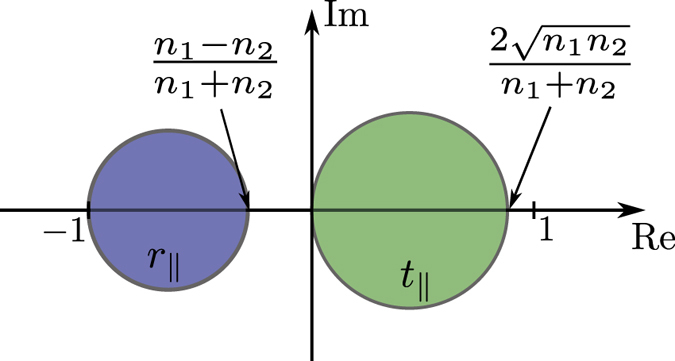
Accessible regions of the complex plane for the transmission and reflection coefficients of a metasurface for *n*_2_ > n1.

The inequalities (19) and (20) limit the transmission and reflection coefficients to two circles in the complex plane as shown in Fig. 2. As we can see from Fig. 2, the phase of the transmission coefficient is limited to the (−*π*/2, *π*/2) interval. Therefore, a transmissive ultrathin metasurface cannot provide full control over the phase of the transmitted light which has the same polarization as the incident light. A special case of this result for a lossless and reciprocal metasurface with *n*_1_ = *n*_2_ is previously presented in ref. 18. When *n*_1_ ≤ *n*_2_ (i.e. the light is incident from the material with the lower refractive index), the attainable reflection coefficients also only cover a phase range smaller than *π* and therefore an arbitrary reflective phase mask cannot be implemented using a metasurface. When the light is incident from the material with larger refractive index (i.e. *n*_1_ > *n*_2_), the phase of the reflection coefficient might cover the full 2*π* range, but if *n*_1_ and *n*_2_ are of the same order of magnitude then the reflection efficiency cannot be large for all phases. For example, if the ultrathin metasurface is fabricated on a silicon substrate with refractive index of *n*_1_ = 3.48 and cladded with air, then the reflection efficiency of an infrared light which is incident from the silicon side and is reflected with zero phase is smaller than (*n*_1 _−_ _*n*_2_)^2^/(*n*_1_ + *n*_2_)^2^ = 31%.

### Polarization Manipulation using Ultrathin Metasurfaces

Ultrathin metasurfaces can also be designed to modify the polarization of an incident light. Polarization modification is achieved by converting the polarization of all or part of the incident light to a polarization orthogonal to that of the incident light. For such metasurfaces, by using (12)–(14) we obtain


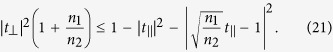


The right hand side of (21) can be simplified further as


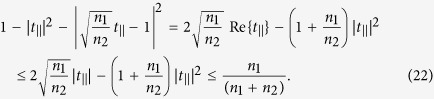


The left hand side of the last inequality in (22) is a quadratic function of |*t*_||_|, and the right hand side of the inequality represents the maximum of this quadratic function. By combining (21) and (22) we find the limit on the polarization conversion efficiency of the transmitted light as


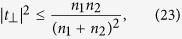


and by using (14) we obtain the limit on the polarization conversion efficiency in reflection as


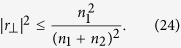


**Figure 3:**
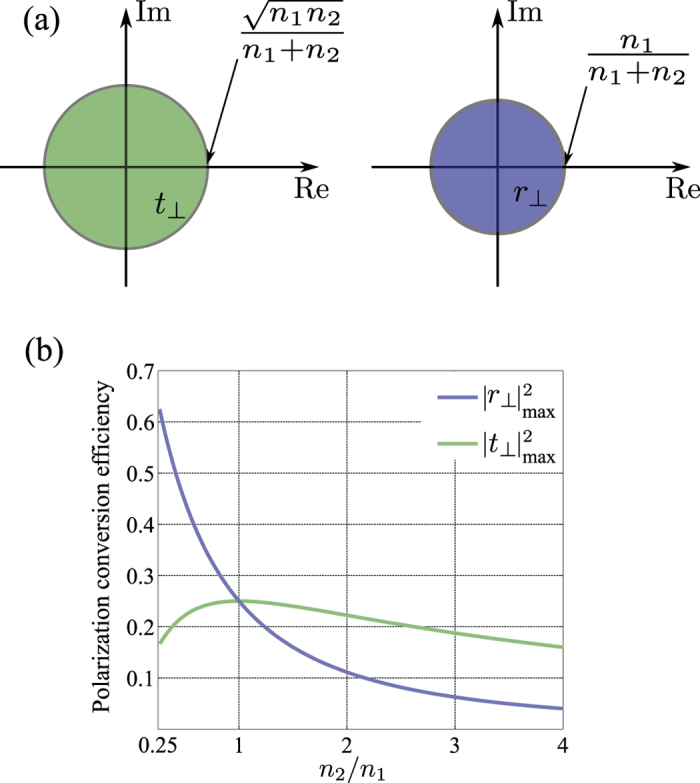
(**a**) Regions of the complex plane admissible for the polarization conversion coefficients *t*_⊥_ and *r*_⊥_. (**b**) Maximum polarization conversion efficiency for a transmissive and reflective ultrathin metasurface as a function of the ratio of the refractive index of the substrate to that of the cladding.

The inequalities (23) and (24) limit the performance of a metasurface designed to manipulate the light’s polarization. For example, the maximum efficiency of a transmissive metasurface half-wave plate which rotates the polarization of a linearly polarized beam by 90 degrees, and is fabricated on a fused silica substrate with the refractive index of *n*_2_ = 1.44 is about 24%. As another example, a transmissive metasurface quarter-wave plate fabricated on the fused silica substrate cannot be more than 48% efficient in converting the polarization from linear to circular. Similar limits can be established for efficiencies of any metasurface that modifies the polarization of light. We note that (23) reduces to |*t*_⊥_|^2^ = 0.25% which has been previously shown for a lossless and reciprocal metasurface when *n*_1_ = *n*_2_^18^. We note that this limit is tight, and polarization conversion efficiencies approaching the limit have been recently reported^21^. Figure 3(a) shows the values in the complex plane that *t*_⊥_ and *r*_⊥_ can achieve. As this figure shows, the phases of *t*_⊥_ and *r*_⊥_ may take any values in the (0, 2*π*) interval, but the transmission and reflection efficiencies are limited to the values enforced by (23) and (24), respectively. Therefore, a limited efficiency transmissive phase mask which imposes an arbitrary phase profile might be implemented using a metasurface if we allow the polarization of the phase shifted transmitted light to be orthogonal to that of the incident light. Such metasurfaces which implement phase masks corresponding to a lens and an axicon have been previously reported^5^.

The maximum polarization conversion efficiencies for a metasurface as a function of the ratio of the substrate to cladding refractive indices have been plotted in Fig. 3(b). As this figure shows, a single transmissive metasurface cannot change the polarization with an efficiency more than 25%. However, the efficiency of a single layer reflective metasurface can be higher if the light is impinging on the metasurface from the higher index material.

## Discussion

The approximation of the local transmission and reflection coefficients of a gradient metasurface with those of a periodic one composed of the same shape scatterers has been successfully used in many metasurface designs reported so far. Although we have also used this assumption here, its accuracy is not essential in the derivation of the limits because we do not use this assumption directly. The main assumption used is the locality of the scattering from the metasurface which allows for defining local transmission and reflection coefficients and the local conservation of energy (i.e. (6)). We also note that, because of the continuity of the tangential electric field at the metasurface interface, nonlocal ultrathin metasurfaces also scatter light symmetrically (i.e. have equal scattered electric field amplitudes) in the substrate and cladding; however, the results presented here are expressed in terms of local transmission and reflection coefficients and cannot be directly applied to them.

The limitations we presented here are results of the non-directionality of the scattering by the ultrathin metasurfaces. In other words, the scattered electric field of an ultrathin metasurface is equal in both the substrate and the cladding layers. Therefore, to overcome these limitations, it is essential to break this symmetric scattering. From (3), we see that the asymmetric emission can only be achieved if the anti-symmetric term (which corresponds to an effective magnetic current) becomes comparable to the symmetric term. For this to happen, 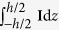 should be smaller than 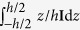 which requires the phase of **I** and thus **J**_e_ to vary at least by *π* as a function of *z* within the thickness of the metasurface. As we discussed, this cannot happen for a single layer ultrathin metasurface. However, a multilayer metasurface might be designed such that the phase of **I** varies among different layers. This has been shown in the microwave regime^16^,^22^,^23^,^24^,^25^,^26^, and more recently, at optical wavelengths^18^,^27^,^28^,^29^,^30^. Nevertheless, since the anti-symmetric term is proportional to *k*_1_*h*, for it to become comparable to the symmetric term, large values of effective current density are required which lead to large absorption losses. This is in agreement with the operation of the multilayer metasurfaces close to resonances where the values of the effective current density are large. We also note that the presented limits are valid for local reflection and transmission coefficients of an interface containing a single layer metasurface, and optical response of a structure containing multiple cascaded interfaces, in general, is not bound by these limits and can be computed using well-known techniques such as the transfer matrix method^31^.

Another approach for overcoming the ultrathin metasurface limits is to use thicker scatterers. Scatterers with a thickness on the order of a wavelength may scatter the light with unequal electric fields into the substrate and cladding layers; therefore, their operations are not limited by the fundamental ultrathin metasurface relations. If we use lossy materials such as gold or silver then thick scatterers absorb significantly, and the absorption limits the efficiency of such metastructures^32^. In this case, the solution is to replace the metal with a low loss dielectric. Indeed, achieving performances beyond the ultrathin metasurface limits have been recently achieved using 1D high contrast gratings^33^,^34^ and high contrast transmitarrays^35^,^36^,^37^,^38^,^39^,^40^,^41^,^42^,^43^,^44^,^45^,^46^,^47^,^48^,^49^,^50^,^51^. High contrast transmitarrays are composed of an array of two dimensional dissimilar dielectric scatterers with the thickness on the order of a wavelength which are arranged on a periodic lattice. Highly efficient phase and polarization control in transmission^40^,^41^,^44^,^47^ and reflection^43^ without altering the polarization (which are fundamentally unachievable using ultrathin metasurfaces) have been experimentally demonstrated.

## Conclusion

We showed that the local transmission, reflection and polarization conversion coefficients of a non-magnetic passive ultrathin metasurface satisfy a set of fundamental relations. These fundamental relations enforce some theoretical limitations on the complex values of these coefficients. We demonstrated that these theoretical limitations are the direct results of the lack of significant phase retardation during the light propagation through the metasurface layer. We can use two approaches to overcome the limitations of ultrathin single layer metasurfaces: cascading ultrathin metasurfaces with other ultrathin metasurfaces, or using high contrast transmitarrays. The latter approach not only is not restricted by the ultrathin metasurface fundamental relations but also does not suffer from the material absorption loss.

## Additional Information

**How to cite this article:** Arbabi, A. and Faraon, A. Fundamental limits of ultrathin metasurfaces. *Sci. Rep.* **7**, 43722; doi: 10.1038/srep43722 (2017).

**Publisher's note:** Springer Nature remains neutral with regard to jurisdictional claims in published maps and institutional affiliations.

## Supplementary Material

Supplementary Information
