# Supplementary material for: Fundamental limits of ultrathin metasurfaces
Source: Sci Rep. 2017 Mar 6;7:43722. doi: 10.1038/srep43722 (PMC5338019; doi:10.1038/srep43722)
Supplement: Supplementary Information [file srep43722-s1.pdf]

Supplementary Information for

Fundamental limits of ultrathin metasurfaces

Amir Arbabi and Andrei Faraon

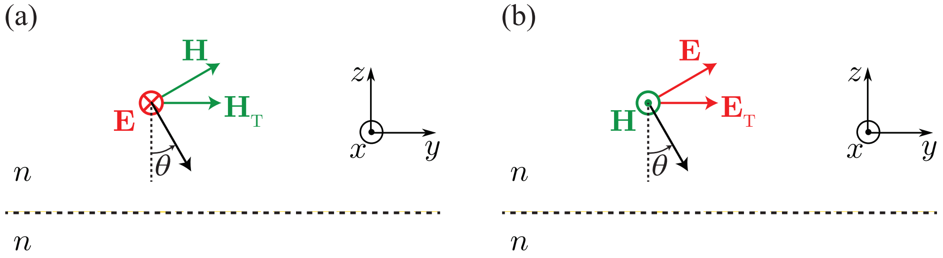

Supplementary Figure 1. Electric and magnetic fields of (a) TE-polarized, and (b) TM-polarized plane waves propagating in a uniform material with a refractive index n and impinging on the xy plane at an angle θ.

RELATION BETWEEN REFLECTION AND TRANSMISSION COEFFICIENTS OF AN ULTRATHIN METASURFACE

Here we present the derivations of equations (15) to (18) of the main text which relate the reflection and transmission coefficients of an ultrathin metasurface for TE and TM polarizations, respectively. To this end, we first relate the reflection and transmission coefficients to the electric fields of reflected and transmitted plane waves. We consider a plane wave that propagates through a homogeneous material with the refractive index n, and is incident at an angle θ on the xy plane (as shown in Supplementary Fig. 1). The optical power per unit area that passes through the xy plane is given by

$$P = \frac{1}{2}(\mathbf{E}\times\mathbf{H}^*)\cdot(-\hat{z}) = \begin{cases} \frac{1}{2}(\hat{z}\times\mathbf{H}^*)\cdot\mathbf{E} = \frac{n}{2Z_0}\cos(\theta)|\mathbf{E}_\mathrm{T}|^2 & \text{for TE polarized plane waves,} \\ \frac{1}{2}(\mathbf{E}\times\hat{z})\cdot\mathbf{H}^* = \frac{n}{2Z_0\cos(\theta)}|\mathbf{E}_\mathrm{T}|^2 & \text{for TM polarized plane waves,} \end{cases} \tag{S1}$$

where $\mathbf{E}_\mathrm{T}$ is the component of the electric field tangent to the metasurface plane (i.e. the projection of the electric field in the xy plane). In deriving (S1), we have also used the relation between the amplitude of the electric and magnetic fields of a plane wave ($|\mathbf{E}| = Z_0|\mathbf{H}|$). In the following, for the sake of brevity, we drop the subscript T with the implicit assumption that all the electric field symbols represent components of their corresponding fields tangent to the metasurface plane. Using (S1), we define the reflection and transmission coefficients that relate the power amplitudes

of plane waves reflected and transmitted from a uniform metasurface as

$$t_{\parallel} = \sqrt{\frac{n_2\cos(\theta_r)}{n_1\cos(\theta_i)}}\frac{\mathrm{E_{t_0}}+\mathrm{E_{s\parallel}}}{\mathrm{E_i}}, \tag{S2}$$

$$r_{\parallel} = \frac{\mathrm{E_{r_0}}+\mathrm{E_{s\parallel}}}{\mathrm{E_i}}, \tag{S3}$$

$$t_{\perp} = \sqrt{\frac{n_2\cos(\theta_r)}{n_1\cos(\theta_i)}}\frac{\mathrm{E_{s\perp}}}{\mathrm{E_i}}, \tag{S4}$$

$$r_{\perp} = \frac{\mathrm{E_{s\perp}}}{\mathrm{E_i}}, \tag{S5}$$

for the TE polarization, and as

$$t_{\parallel} = \sqrt{\frac{n_2\cos(\theta_i)}{n_1\cos(\theta_r)}}\frac{\mathrm{E_{t_0}}+\mathrm{E_{s\parallel}}}{\mathrm{E_i}}, \tag{S6}$$

$$r_{\parallel} = \frac{\mathrm{E_{r_0}}+\mathrm{E_{s\parallel}}}{\mathrm{E_i}}, \tag{S7}$$

$$t_{\perp} = \sqrt{\frac{n_2\cos(\theta_i)}{n_1\cos(\theta_r)}}\frac{\mathrm{E_{s\perp}}}{\mathrm{E_i}}, \tag{S8}$$

$$r_{\perp} = \frac{\mathrm{E_{s\perp}}}{\mathrm{E_i}}, \tag{S9}$$

for the TM polarization. We note that, similar to the normal incident case, the tangential component of the electric field emitted by the metasurface ($\mathrm{E_s}$) is continuous at the substrate-cladding interface. Using (S2) to (S5) and the the continuity of the tangential components of the electric field at a bare interface ($\mathrm{E_{r0}}+\mathrm{E_i}=\mathrm{E_{t0}}$), we find

$$r_{\parallel} = \sqrt{\frac{n_1\cos(\theta_i)}{n_2\cos(\theta_r)}}t_{\parallel} - 1, \tag{S10}$$

$$r_{\perp} = \sqrt{\frac{n_1\cos(\theta_r)}{n_2\cos(\theta_i)}}t_{\perp}, \tag{S11}$$

for the TE polarization. Similarly, using (S6) to (S9) find

$$r_{\parallel} = \sqrt{\frac{n_1\cos(\theta_r)}{n_2\cos(\theta_i)}}t_{\parallel} - 1, \tag{S12}$$

$$r_{\perp} = \sqrt{\frac{n_1\cos(\theta_i)}{n_2\cos(\theta_r)}}t_{\perp}. \tag{S13}$$

for the TM polarization.
